# Effect of Core Mass and Alloy on Cyclic Fatigue Resistance of Different Nickel-Titanium Endodontic Instruments in Matching Artificial Canals

**DOI:** 10.3390/ma14195734

**Published:** 2021-10-01

**Authors:** Sebastian Bürklein, Lennart Zupanc, David Donnermeyer, Karsten Tegtmeyer, Edgar Schäfer

**Affiliations:** 1 Albert-Schweitzer-Campus 1, Central Interdisciplinary Ambulance in the School of Dentistry, Westphalian Wilhelms-University, Building W 30, 48149 Münster, Germany; eschaef@uni-muenster.de; 2 Private Office, Markt 4, 48291 Telgte, Germany; L.Zupanc@gmx.de; 3 Albert-Schweitzer-Campus 1, Department of Periodontology and Operative Dentistry, Westphalian Wilhelms-University, Building W 30, 48149 Münster, Germany; David.Donnermeyer@ukmuenster.de (D.D.); Karsten.Tegtmeyer@ukmuenster.de (K.T.)

**Keywords:** artificial canal, austenitic nickel-titanium, cross section, body temperature, dynamic fatigue tests, fracture resistance, heat treatment, martensitic NiTi, R-phase NiTi, SE-NiTi

## Abstract

Instrument failure during root canal preparation is still a concern among endodontists. However, it remains unclear whether the use of more martensitic alloys or the cross-sectional design parameters (i.e., core mass) significantly improve fracture resistance. The aim of the study was to evaluate the impact of core mass and alloy on dynamic cyclic fatigue resistance of nickel-titanium endodontic instruments in matching artificial canals at body temperature. Two groups were tested. (A) taper 0.04: F360 (Komet, Lemgo, Germany), Twisted file (Sybron Endo, Glendora, CA, USA) (=TF), JIZAI (Mani, Tochigi, Japan) (=J_04) (all size #25) and the variable tapered TruNatomy (Dentsply, Ballaigues, Switzerland) (size #26) (=TN). (B) size #25; taper 0.06: (Mtwo (VDW, Munich, Germany), JIZAI (Mani) (=J_06), and variable tapered Hyflex EDM OneFile (Coltene Whaledent, Altstätten, Switzerland) (=HF). Time, number of cycles to fracture (NCF), and number and length of fractured fragments were recorded and statistically analysed using ANOVA Student-Newman-Keuls, Kruskal–Wallis or Chi-square test (significance level = 0.05). (A) TN showed the significantly shortest time until fracture, followed by TF, F360 and J_04 which also differed significantly, while NCF showed the following order: F360 < TN < TF < J_04 (*p* < 0.05). Only one J_04 but all instruments of the other groups fractured within the test-limit of 10 min. (B) Mtwo was significantly inferior concerning time until fracture and NCF, compared to J_06 and HF (*p* < 0.05), which did not differ significantly (*p* > 0.05). While all Mtwo instruments fractured, only four instruments failed in the other groups (*p* < 0.05). Within the limitations of this study, alloy and cross-sectional design (i.e., core mass) were critical factors regarding instrument failure, but none of these factors could be determined as a main parameter for increased or decreased time, and cycles to fracture. Rather, it seemed to be the interaction of multiple factors (e.g., longitudinal and cross-sectional design, alloy, and rotational speed) that was responsible for differences in the time and cycles to fracture. Nonetheless, all instruments had lifetimes that allow safe clinical use. However, the superiority or inferiority of an instrument with regard to cyclic fatigue based on laboratory results—even when identical trajectories are guaranteed—may be considered questionable, as the characteristics and design parameters of the instruments vary considerably, and the experimental setups lack additional clinical parameters and thus clinical relevance.

## 1. Introduction

The steadily growing number of publications concerning cyclic fatigue resistance of nickel-titanium (NiTi) endodontic root canal instruments indicates that this topic is of interest among endodontists. Although the prevalence of instrument fracture is relatively low, it is an adverse event for both the patient and the practitioner [1,2,3,4,5,6]. The prevalence for instrument fracture depends on study design and instruments, and varies between 0.3 and about 7% of cases, and ranges below 1–2% for the instruments used [1,2,3,4,5,6].

Many reasons can promote a fracture of NiTi instruments. Tooth anatomy and root canal morphology with the associated angles and radii of curvatures are known as influencing factors [7]. Furthermore, instrument-associated parameters such as alloy, manufacturing process, cross-sectional design, taper and core mass are also important parameters [8]. Concomitant factors, such as the number of uses and the number of sterilization processes [9], the irrigation solutions used [10], and the kind of movement (reciprocal vs. rotary), vertical amplitude, axial forces and rotational speed [11,12], are also discussed. Instruments are stressed during active root canal preparation and may fracture due to cyclic fatigue, torsional load, or due to a combination of both [13,14]. However, cyclic fatigue is claimed to be the main cause for failure of endodontic engine-driven NiTi instruments [15,16]. 

Fracture of an endodontic instrument may exert an impact on the prognosis of root canal treatment when infected root canal areas are no longer accessible and maintain persistent inflammation [17,18]. Hence, when an accidental fracture occurs or is occasionally detected on a routine radiograph, the patient should be informed [19]. Treatment options are limited to retaining the fragment inside the root canal as a metallic obstruction/root canal filling, bypassing or removing it. The decision making process should be based on (i) the stage of the root canal treatment procedure at which the instrument fractured, (ii) the clinician’s skills and equipment, (iii) the potential risks of iatrogenic damage of tooth structure when removing the fractured instrument, (iv) the length and the location of the fractured fragment in the root canal, and (v) the presence or absence of apical periodontitis [20] 

Although cyclic fatigue is not currently included in ISO-specification 3630-1 [21], instrument resistance has some clinical relevance, as instruments working time may represent an important parameter. One major limitation of laboratory studies is the lack of standardization, and many studies do not mention all relevant details of experimental settings [22]. Hence, results differ widely and are hardly comparable [22]. A crucial aspect seems to be the congruence between the artificial canal wall and the dimensions of the instrument [23]. A recent publication elucidated the influence of artificial root canal size that should be as close as possible to the size of the instrument within the manufacturer’s tolerance—meaning a matching size [24]. Apart from that, dynamic testing at body temperature with a vertical amplitude of 3 mm and a frequency of 0.5 Hz may be regarded as a kind of standard performing dynamic cyclic fatigue test [22]. 

Currently, no studies on cyclic fatigue resistance are available investigating the parameters core mass and alloy in matching artificial root canals. Thus, the aim of this study was to investigate the impact of core mass and alloy of different endodontic NiTi instruments with the same outer diameter and different cross-sectional designs on cyclic fatigue using matching artificial tubes. The null hypothesis (H0) tested was that the two parameters have no impact on the fracture resistance of the tested instruments.

## 2. Materials and Methods

The experimental setup presented in the following aimed to evaluate cyclic fatigue of different engine-driven NiTi instruments with different designs, tapers and sizes using standardized matching artificial root canals guaranteeing same trajectories. 

### 2.1. Sample Size Calculation

Prior to the evaluation of the main study, a preliminary investigation under identical conditions was performed using five instruments in each group. Mean values and standard deviations revealed an effect size of >1.0. A markedly lower effect size of 0.4 served for calculation using G*Power 3.1 (Heinrich Heine University, Düsseldorf, Germany). Using the parameters of an alpha (α) level of 0.05 (5%) and a (β) beta level of 0.20 (20%) (i.e., power = 80% at a 5% significance level) and an effect size of 0.4, the sample size was 19 in each group. Thus, 20 instruments in each group were used (total = 120).

### 2.2. Artificial Canals

According to a previous study, artificial root canals matching the dimensions of the instruments investigated were designed using Geomagic Freeform software (3D Systems, McLean, VA, USA) [24]. 

**Figure 1:**
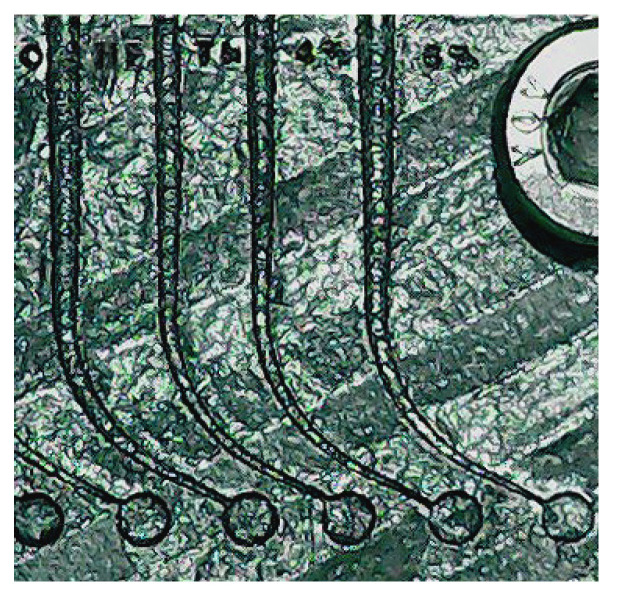
Artificial root canals produced in an additive laser melting process from the left to the right—Hyflex OneFile tube (25/~ variable tapered), TruNatomy (26/0.04 variable tapered), 25/0.04, 25/0.06. All tubes had a diameter corresponding to the instrument +0.02 mm to guarantee a friction-free free rotation without any additional heat development.

Four different canals with instrument size plus 0.02 mm in diameter served as simulated root canals. The artificial canal size 27/0.04 was used to test all instruments having a size of 25/0.04: F360 (Komet, Lemgo, Germany), Twisted file (Sybron Endo, Glendora, CA, USA) (=TF), JIZAI (Mani, Tochigi, Japan) (=J_04). The TruNatomy (Dentsply, Ballaigues, Switzerland) (=TN) instrument has a variable taper over its working part and starts with a size of 26/100 mm (Figure 1). The artificial canal was made using the instrument parameters specified by the manufacturer, which were additionally checked millimetre by millimetre several times using a digital calliper by adding 0.02 mm. Hence TN started at 0.28 mm. In the group with taper 0.06 the artificial canal offered a size of #27/0.06 for Mtwo and JIZAI (=J_06), whereas for the variable tapered Hyflex (=HF) the artificial canal was prepared in the same manner as described for TN. 

All artificial canals had a 60° curvature and a 5 mm radius, with the centre of curvature at 5 mm from the apex and were 16 mm long, corresponding to the working part of the instruments. Canals terminated in a circle at their end simulating an open system and allowing a kind of reservoir and refreshing of the lubricant used. The designed STL data of artificial canals were transferred into a CoCr model using a special digital metal laser sintering (DMLS) method (Infinident Solutions, Darmstadt, Germany). This is a unique additive manufacturing process based on a cobalt-based metal ceramic alloy. The material (fine-grain powder) was applied on a panel by a so-called re-coater, and a 200 W Yb (ytterbium) fibre laser melted the material layer by layer using shielding gas (nitrogen (N2)). The whole process was repeated by a predefined layer thickness of 20–40 µm that was established by lowering the panel accordingly. A thermal post-processing procedure served for stress-relief annealing. Finally, the material was cleaned by sandblasting. The size of the artificial canals was measured by a laser scanning microscope (Keyence VK-X1000, Keyence Corporation, Osaka, Japan) to verify the agreement between the actual and the target sizes.

### 2.3. Cyclic Fatigue Testing 

**Figure 2:**
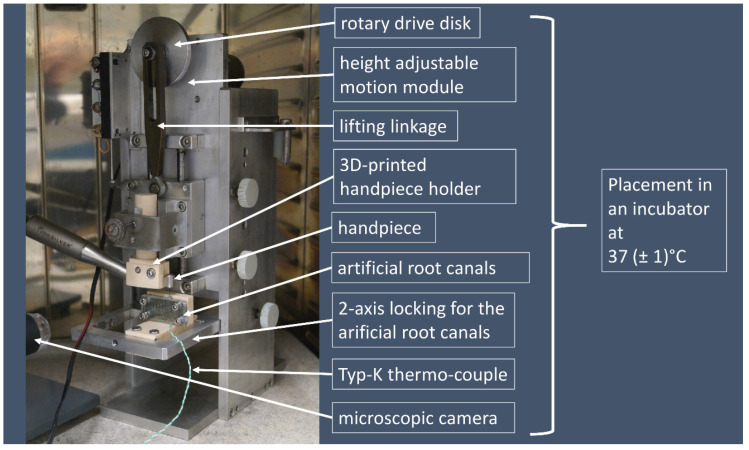
Setup of the cyclic fatigue testing device placed in an incubator. Tests were recorded with a microscopic camera.

The complete experimental setup was placed in an incubator (SFM500, Memmert, Schwabach, Germany) (Figure 2) guaranteeing maintenance of body temperature (37 ± 1 °C) throughout the entire procedure. Additionally, the temperature inside the artificial canal was checked permanently using thermo-K couples (GHM, Messtechnik, Regenstauf, Germany) with a frequency of 50 Hz (Figure 2).

The artificial canals were covered with tempered glass to prevent the instruments from slipping out of the tube [24]. Glycerin oil warmed to body temperature served as a lubricant and was continuously refreshed during the tests to avoid any friction. It was applied through a small window in a Plexiglas plate that served as the door of the incubator. 

All instruments were tested according to manufacturer’s instructions—rotational speed and torque settings were set to the demanded values using a torque controlled endodontic motor (VDW silver, VDW, Munich, Germany): 

F360 (25/0.04) = 300 rpm, 1.8 Ncm

Twisted file (25/0.04) = 500 rpm, 2.5 Ncm

JIZAI (J_04) (25/0.04) = 500 rpm, 3.0 Ncm

TruNatomy (26/0.04; variable tapered; 0.04 in average) = 500 rpm, 1.5 Ncm

Mtwo (25/0.06) = 280 rpm, 2.3 Ncm

JIZAI (J_06) (25/0.06) = 500 rpm, 3.0 Ncm

Hyflex Onefile (25/~; variable tapered; not specified) = 400 rpm, 2.5 Ncm

To simulate the dynamic up and down movements (picking movement, stroking) during root canal treatment, an eccentric mount 1.5 mm from the central axis (total amplitude = 3 mm) was used, which was fixed on a continuously rotating drive disk (Figure 2). The speed was set to 1 cycle/2 s (=0.5 Hz).

A 3D printed and assembled handpiece holder, especially fabricated for the present study served for a correct positioning of the handpiece (Figure 2). The test machine allowed a free adjustment of the different components to each other.

The tests were recorded with a microscopic camera using the AMcap software (AMcap, version 3.0.1.7; NCH Software, Greenwood, CO, USA). The automatic lifting device and the endodontic motor were started simultaneously. When an instrument fractured, the testing machine and then the video were stopped. The final temperature was noted in the experimental protocol and the video was saved. 

The start and end time of the experiment were determined based on the video recording (Movies and TV App, Microsoft Corporation, Redmond, WA, USA, version 10.20112.10111.0). The start time was defined as the moment when the endodontic instrument first reaches the working length. The end time was defined as the time at which the instrument fractured. The tests were limited to 600 s. Unless a cyclic fatigue fracture occurred within 600 s., the test was terminated with a buffer time of 30 s. 

The cycles to fracture were determined based on the time of instrument failure:

(1) $$NCF=\frac{\mathrm{time}\;\mathrm{to}\;\mathrm{fracture}}{60}\times rpm$$

*NCF* = number of cycles to fracture; *rpm* = rotations per minute

### 2.4. Analysis of the Fractured Instruments

The lengths of the fractured fragments were measured with a digital calliper (Mitutoyo 500-196-30 Absolute AOS Digimatic; Mitutoyo Corporation, Kawasaki, Japan) and the values were noted.

To check the fracture mode, five randomly selected fragment surfaces in each group, if available, were examined using a laser scanning microscope (Keyence VK-X1000, Keyence Corporation, Osaka, Japan) at a 500-fold magnification to exclude a torsional fracture. For the examination, the fracture surfaces were cleaned with 70% alcohol and embedded in a silicone block.

### 2.5. Statistical Analysis

Statistical analysis was performed using SPSS 20 software (SPSS Inc., Chicago, IL, USA). The obtained data were checked for normal distribution using Kolmogorov–Smirnov and Shapiro–Wilk tests. Normally distributed data were analysed using the ANOVA Student-Newman–Keuls (SNK) test. Non-normally distributed values were evaluated using the Kruskal–Wallis test. Chi-square test served for statistical analysis of the fracture mode. The significance level was set at *p* < 0.05. 

## 3. Results

The results listed hereafter address the time and cycles to instrument fracture as well as the length of the fracture fragments (Table 1, Table 2, Table 3 and Table 4). Additionally, a fractographic laser scanning microscopic examination (Keyence VK-X1000) of the fracture surfaces was performed.

### 3.1. Time and Cycles to Fracture—Taper 0.04

The time to fracture and the number of cycles to fracture are listed in Table 1 and Table 2. Time to fracture differed significantly between all instruments in the following order from shortest to longest time: TN < TF <F360 < J_04 (*p* < 0.05), whereas cycles to fracture showed a different order: F360 < TN < TF <J_04 (*p* < 0.05). Only one instrument in the J_04-group, but all instruments in the other groups, fractured (*p* < 0.05).

### 3.2. Time and Cycles to Fracture—Taper ≥ 0.06

None of the Mtwo instruments reached the maximum testing time, whereas 16 instruments in the Hyflex- and the J_06-group exceeded the end of testing time (*p* < 0.05). Regarding the time and cycles to fracture HF and J_06 performed significantly better compared to Mtwo (*p* < 0.05). 

### 3.3. Fracture Lengths—Taper 0.04

The results of the fragment lengths also showed statistically significant differences between the instrument groups (*p* < 0.05) (Table 3). The TruNatomy instrument showed the shortest mean fragment lengths that was statistically significantly different from the F360 and Twisted File (*p* < 0.05). The JIZAI_04 instrument, with a mean fragment length of 2.70 mm, did not differ from the other groups (*p >* 0.05).

### 3.4. Fracture Lengths—Taper > 0.06

The test results of the fragment lengths of the 0.06 or larger tapered instruments showed statistically significant different values (*p* < 0.05) (Table 4). Hyflex OneFile instrument with a length of 5.02 mm significantly exceeded the other two instruments (*p* < 0.05), which did not differ from each other (*p* > 0.05). 

### 3.5. Cross-Sectional Parameters of the Instruments

**Figure 3:**
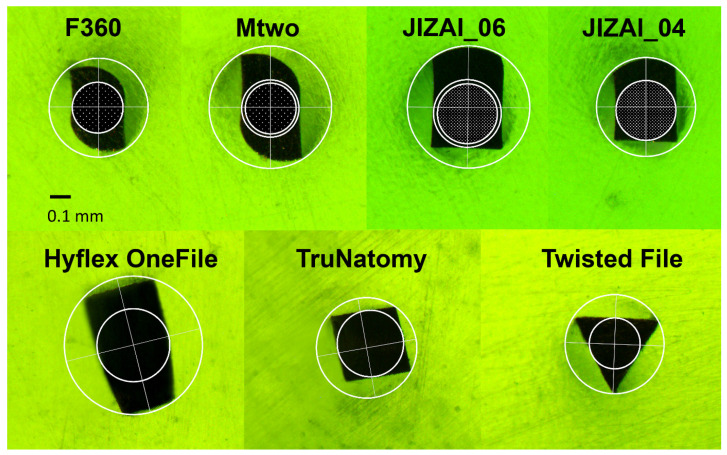
Cross sections of the different endodontic instruments at 6 mm from the tip. The upper row shows the instruments with identical cross-sections but different sizes. The increase of the metal mass of instruments with the same cross-sectional design due to the greater taper is visualized by the dotted circles projected into the larger instruments.

All instruments varied concerning the parameter listed in Table 5. Despite of equal (outer) diameter sizes in the 0.04 tapered and almost equal sizes in the greater tapered (≥0.06) group, core diameters and cross-sectional heights differed widely due to the specific instrument designs (Figure 3). Instruments with an identical cross section (F360 & Mtwo = S-shaped; JIZAI 04 & 06 = eccentric slender rectangular with radial lands) offered an increase of the core diameter of about 13% with a change of the taper from 0.04 to 0.06 resulting in a different width-height ratio at the same section of the instruments (6 mm from its tip) (Figure 3). 

### 3.6. Analysis of the Fractured Instruments

**Figure 4:**
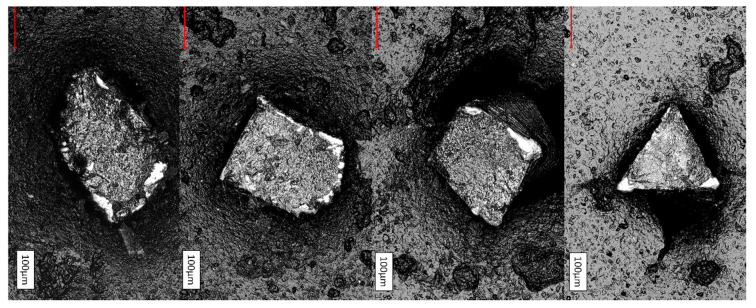
Exemplary laser scanning microscope views of 0.04-tapered instrument fracture surfaces. F360, JIZAI_04, TruNatomy, Twisted File. The white areas represent the crack initiation. All fracture modes were characteristic for “cyclic fatigue”.

**Figure 5:**
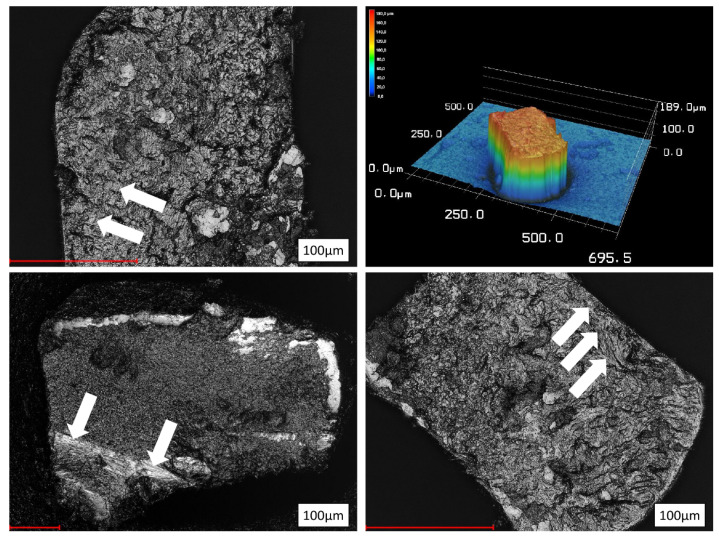
Fracture surfaces of 0.06 tapered instruments or larger size. Left side: Mtwo (**top**) and Hyflex OneFile (**bottom**); arrows indicate the typical striation areas of crack propagation; Right side: JIZAI_06 in a 3D-surface relief with corresponding data (µm) and laser scanning surface view at high magnification.

All fracture surfaces showed typical signs of cyclic fatigue (Figure 4). Fatigue propagation areas revealed striations (Figure 4 and Figure 5). Additional large, ripped portions were present as signs for catastrophic failure caused by the continuously reduced core mass due to cyclic stress. According to the instruments cross-sectional designs, crack growth regions differed in size and the region of origin.

## 4. Discussion

The present results showed significant differences between the tested instruments concerning time to fracture, NCF and lengths of fractured fragments. Thus, the null hypothesis (H0) must be rejected, as core mass and alloy influenced the fracture strength of the endodontic instruments subjected to dynamic cyclic fatigue testing.

### 4.1. Core Mass and Cross-Sectional Design

The impact of the cross-sectional design and the core mass on cyclic fatigue resistance has already been shown and a smaller metal volume (core mass) seems to be associated with greater resistance to cyclic fatigue [25,26,27]. When calculating the bending section modulus $Z=\frac{I}{y}$ with the according parameters I = second moment of area (moment of inertia); y = distance from the neutral axis to any given fibre, impact may get explained by the distance of the neutral axis in the instrument centre to the instrument surface and geometrical shape. For symmetrical sections the value of Z is the same above or below the centroid. Asymmetrical sections offer several different values depending on the cross-sectional design. In recent FE-analysis, cross-sectional design, centricity of the cross-section, taper and pitch revealed to influence bending moments of instruments [28]. This computational evidence has already been supported in laboratory studies. Zhang et al. proved a more pronounced impact of the cross-sectional design compared to taper or size of the instrument on the stresses developed due to different trajectories under load [29]. In general, a flat design of instruments (slender rectangular; S-shape) seems to favour increased cyclic fatigue resistance [30]. 

The instruments investigated in the present study offered different cross-sectional designs. Whereas Hyflex OneFile has a varying cross-sectional design from the tip region to its shaft, from rectangular to trapezoidal to triangular, the other instruments possess the same cross-sectional design over their entire working part: TruNatomy (rectangular, square), Twisted file (triangular cross section), Mtwo and F360 (S-shape), JIZAI (modified slender rectangular eccentric cross section with a radial land) (Figure 3, Figure 4 and Figure 5). 

The present study elucidated that the core diameter was not decisive for the longevity of the tested instruments. Core size/mass did not correlate with time to fracture or NCF (Table 1, Table 2 and Table 5). Neither did the width-to-height ratio affect the results (Table 5). The difference to the above-mentioned studies may be explained by the variabilities in the experimental setups. Under clinical conditions, instruments must engage into the dentinal walls to remove dentin and thus, generation of load is a much more complex process. Torsional, axial and lateral forces also provide for stress. Apart from that root canal anatomy and the specific instrument design are considered being responsible for screw-in forces and instrument load [31,32,33] 

Two instruments (J_04 and J_06) of the same manufacturer offering both identical cross-sectional design and alloy were tested in this study. Additionally, F360 and Mtwo (different manufacturers) possess an identical S-shaped cross-sectional design and the same alloy with comparable metallurgical properties. This allows a comparison of NCF and time to fracture in matching artificial canals of identical instruments with an identical experimental setup. Whereas JIZAI instruments also had identical rpm and torque values, the two other instruments differed concerning motor settings (300 vs. 280 rpm and 1.8 vs. 2.3 Ncm). 

Both Mani instruments exceeded the maximum testing time in the majority of the cases. Failure rate (J_04 = one instrument; J_06 = four instruments) did not differ significantly. Nevertheless, results show a tendency of a reduced working time using larger instruments (482 sec. vs. 559). Due to the small number of failures a statistical analysis of the time values was not possible. It has to be taken into consideration that at the same section, both the core mass and the width-height ratio differ in JIZAI instruments with different tapers (taper 04 vs. taper 06)—1.4 vs. 1.5, respectively. 

When comparing F360 and Mtwo (identical S-shaped cross-sectional design), the bigger instrument showed a significantly longer life span, but NCF was almost the same. This may be explained by the different rotational speeds. Hence, both the core diameter and the outer diameter were not solely decisive for cyclic fatigue resistance of endodontic NiTi instruments. 

### 4.2. Alloy 

Thermomechanical processing of endodontic instruments is used to optimize the microstructure and transformation behaviour of NiTi alloys [34]. SE-NiTi, R-Phase and other heat-treated alloys offer different Austenit finishing (A_f_) temperatures that influence instrument properties during root canal preparation at body temperature. Whereas SE-NiTi is mainly austenitic at clinically relevant temperatures, the heat-treated NiTi instruments show more ductile martensitic characteristics. Currently available studies show an increased cyclic fatigue resistance of heat-treated endodontic instruments [35,36,37]. Instruments used in the present study were made of SE-NiTi (F360 and Mtwo), R-Phase (Twisted file), Gold-wire (TruNatomy) and CM wire/EDM (electric discharge machining) (Hyflex). The present results indicate that heat treatment did not necessarily lead to an increased fracture resistance. 

Additionally, the manufacturing processes of the instruments differed. Whereas F360, JIZAI, Mtwo and TN were ground out of a blank to get the cross-sectional design, the other two instruments were subjected to unique manufacturing processes. TF is twisted out of a prefabricated triangular blank and HF is manufactured by electric discharge machining (EDM). The main advantage of the latter two manufacturing processes is that these instruments do not show any surface micro defects, which are usually induced by the surface processing. Those instruments are claimed to have extended fatigue resistance due to the absence of surface defects [38]. This finding was corroborated by the present results in a far as in the 0.04-tapered group, TF was superior to the other instruments except JIZAI concerning the NCF. Thus, a beneficial effect of the unique manufacturing process was supported by the present observations. 

### 4.3. Rotational Speed 

Rotational speed seems to have a major impact on cyclic fatigue as increased speed tends to reduce time to fracture [39]. The more rotations per minute the higher the stress and strain rate which is accompanied with a reduced time for relaxation [40]. The influence of heat generation by friction and a possible phase conversion depending on the A_f_-temperature are also discussed [41]. However, in the present study, this aspect seems to be negligible due to the continuous refreshment of the lubricant and the real time measurement of the temperature. The obtained results did not corroborate that time-to-failure for NiTi instruments decreased significantly as rotational speeds increased [42].

However, the NCF may allow comparison of instruments with different rotational speed. From a clinical point of view, the time until fracture is more relevant when conforming to the manufacturer’s instructions as the rotational speed is predetermined. This raises the question of whether time to fracture or NCF is the parameter that should be evaluated in future cyclic fatigue studies. NCF was implemented to compare instruments with different rpm or even working modes (rotary/reciprocating, adaptive). Actually, no evidence-based protocol for the comparison of different working modes is available. The rotational speed employed for any instrument should always be considered in accordance with the manufacturer’s recommendations, the clinical situation and the experience of the operator. 

### 4.4. Experimental Setup

The experimental setup has been presented in detail in a previous study and the advantages and disadvantages have already been discussed [24]. The design was based on a dynamic approach with a 3 mm amplitude and frequency of 0.5 Hz in matching artificial root canals at body temperature. Thus, the most commonly used values were adopted [22]. 

All artificial canals were especially designed according to the instruments sizes and taper. The setup guaranteed identical angles of curvature during testing for all instruments by matching artificial canals that were designed and manufactured using an additive laser melting approach [24]. Thus, all instruments were tested in a standardized manner due to consistent settings and trajectories during testing [24,43,44]. A previous study has shown that as the curvatures of the instruments subjected to cyclic fatigue tests in non-matching artificial tubes were reduced, the looser the instruments fit to the artificial canals. This was explained by the reduced deflection in larger artificial canals [24]. Additionally, as the complete experimental setup was placed in an incubator (Figure 2) and the temperature inside the artificial canal was checked permanently using thermo-K couples it was ensured that intracanal temperatures simulated the clinical situation well [45], because there is evidence that the longevity of instruments in cyclic fatigue tests significantly depends on A_f_ (Austenit finishing) temperature of the alloys [46,47,48,49,50]. Human body temperature varies between 36.3 °C and 37.4 °C. These values are considered normal temperatures. Thus, 37 °C served as a baseline to address the range of human body temperatures consistent with most current laboratory studies [46,47,48,49]. Even though de Hemptinne et al. showed slightly lower intracanal values in vivo [45].

### 4.5. Weaknesses

However, although the present setup aimed to simulate active root canal preparation as accurately as possible, it could not exactly replicate actual clinical conditions. Thus, despite its improved standardization, this study also had inherent weaknesses:

(i) No setup currently used for cyclic fatigue testing can simulate the exact clinical conditions of endodontic treatment procedures with additional axial loading and active dentin removal in a standardized manner.
(ii) Different instruments made of different alloys with different cross-sectional shapes, designs and core masses, as well as rotational speeds are difficult to compare in terms of cyclic fatigue.
(iii) Superiority or inferiority in a laboratory study is not necessarily associated with better or worse performance in the patient. Therefore, the finding of a relationship between individual study parameters and increased fracture resistance of an endodontic instrument does not necessarily indicate causality.

## 5. Conclusions

Within the limitations of the present study, it can be stated that NCF did not reflect the time to fracture well. Heat treatment, alloy, core and outer diameter exert an impact on cyclic fatigue resistance of endodontic NiTi instruments. It can be assumed that unique manufacturing processes such as instrument twisting or electric discharge machining positively influence longevity. However, none of the factors could be determined as a main parameter that necessarily led to an increase or decrease of time and/or cycles to fracture. It seemed impossible to identify special design parameters that were responsible for fracture resistance when testing different instruments with the same outer diameter, but different alloys and cross-sectional geometries, even when guaranteeing almost identical trajectories by an optimized experimental setup. Rather, the interplay of numerous factors (e.g., longitudinal and cross-sectional design, alloy, and rotational speed) in fact appeared to be decisive. Currently used and widespread experimental setups obviously overestimate instruments properties and longevity due to inconsistent and non-reliable trajectories, i.e., when testing the cyclic fatigue of (variable) tapered heat-treated endodontic instruments. Further studies are required to assess the impact of design parameters and the material properties of endodontic instruments with better standardization. No laboratory setup currently succeeds in simulating operator-related parameters and exact clinical conditions, including active cutting action, torsional stress, axial pressure, fed-in-effect, and irrigation solutions. Thus, the available results and data concerning cyclic fatigue tests of endodontic NiTi instruments may be considered questionable and suffer from any lack of clinical relevance. The significance of the results obtained and the usefulness of performing laboratory cyclic fatigue tests should be investigated regardless.

However, clinicians should always be aware of the characteristics and design parameters of instruments used in root canal preparation, as well as their own experience and skills.

## Figures and Tables

**Table 1:** Time to fracture and number of cycles to failure of the 0.04-tapered instruments with standard deviations and min and max values. * Tests were stopped after 10 min (=600 s) if no fracture occurred.

Instrument Taper 0.04	Instruments Fractured	rpm	Time to Fracture (sec) *				Cycles to Fracture			
			Mean	Sd	Min	Max	Mean	Sd	Min	Max
F360	20/20 ^b^	300	211.2 ^c^	60.44	123	304	1055.75 ^a^	302.2	615	1520
TN	20/20 ^b^	500	150.2 ^a^	27.02	114	198	1251.7 ^b^	225.2	950	1650
TF	20/20 ^b^	500	178.7 ^b^	48.11	112	267	1489.2 ^c^	400.9	933	2225
JIZAI_04	1/20 ^a^	500	>600 ^d^	-	559	n/a	>5000 ^d^	-	4658	n/a

Values with different superscript letters were statistically different (normally distributed data = SNK-test, significance level 0.05).

**Table 2:** Time to fracture and number of cycles to failure of the 0.06 or larger tapered instruments with standard deviations and min and max values. * Tests were stopped after 10 min (=600 s) if no fracture occurred.

Instrument Taper 0.06	Instruments Fractured	rpm	Time to Fracture (sec) *				Cycles to Fracture			
			Mean	Sd	Min	Max	Mean	Sd	Min	Max
Mtwo	20/20 ^b^	280	255 ^a^	35.95	198	335	1191 ^a^	154.0	924	1563
Hyflex	4/20 ^a^	400	>600 ^b^	-	353	>600	>4000 ^b^	-	2353	>4000
JIZAI_06	4/20 ^a^	500	>600 ^b^	-	482	>600	>5000 ^c^	-	4017	>5000

Values with different superscript letters were statistically different (normally distributed data = SNK-test, significance level 0.05).

**Table 3:** Fractured instruments in the taper.04 group with standard deviation (sd), min and max values.

Instrument Taper 0.04	Instruments Fractured	Lengths of Fractured Instruments (mm)			
		Mean	Sd	Min	Max
F360	20/20 ^b^	2.95 ^b^	0.35	2.33	3.76
TN	20/20 ^b^	2.29 ^a^	0.23	1.76	2.65
TF	20/20 ^b^	3.04 ^b^	0.56	1.73	4.33
JIZAI	1/20 ^a^	2.70 ^a,b^	-	-	-

Values with different superscript letters were statistically different (Kruskal–Wallis-test, significance level 0.05).

**Table 4:** Fractured instruments in the taper ≥ 0.06 group with standard deviation (sd), min and max values.

Instrument Taper ≥ 0.06	Instruments Fractured	lengths of fractured instruments (mm)			
		Mean	Sd	Min	Max
Mtwo	20/20 ^b^	2.87 ^a^	0.51	2.12	4.03
Hyflex	4/20 ^a^	5.02 ^b^	0.93	3.95	5.62
JIZAI	4/20 ^a^	2.29 ^a^	0.39	1.84	2.63

Values with different superscript letters were statistically different (Kruskal-Wallis-test, significance level 0.05).

**Table 5:** Instrument cross sectional parameters at 6 mm from the tip. (v = variable tapered, 0.04 in average; ~ variable tapered but not specified by the manufacturer).

Instrument Cross-Section Parameters							
Instrument	Size/ Taper	Cross Sectional Design	Centered or Off-Centered	Outer Diameter at 6 mm [mm]	Core Diameter [mm]	Cross Sectional Height [mm]	Approx. Width-Height Ratio
F360	25/0.04	S-shape	centered	0.45	0.23	0.37	1:1.6
TruNatomy	26/0.04 v	Rectangular	off-centered	0.45	0.29	0.30	1:1
Twisted file	25/0.04	Triangular	centered	0.45	0.23	0.34	1:1.5
JIZAI_04	25/0.04	(slender) rectangular	off-centered	0.45	0.27	0.37	1: 1.4
Mtwo	25/0.06	S-shape	centered	0.55	0.26	0.48	1:1.8
Hyflex Onefile	25/~	Trapezoidal	centered	0.62	0.33	0.55	1:1.7
JIZAI_06	25/0.06	(slender) rectangular	off-centered	0.55	0.30	0.45	1:1.5

## Data Availability

The data presented in this study are available on request from the corresponding author.
